# Infection risk factor measurement based on spatial and temporal distribution of COVID-19 nucleic acid test sites: an example from Shenzhen

**DOI:** 10.3389/fpubh.2024.1420497

**Published:** 2024-10-21

**Authors:** Lei Wang, Longhao Zhang, Tianlin Zhang, Xin Han

**Affiliations:** ^1^School of Architecture, Tianjin University, Tianjin, China; ^2^College of Landscape Architecture, Zhejiang A&F University, Hangzhou, China

**Keywords:** COVID-19 nucleic acid detection sites, built environmental factors, infection risk index, policy recommendations, spatial–temporal analysis

## Abstract

COVID-19 has profoundly impacted global daily life, emphasizing the need for effective virus suppression strategies. In response, China has established numerous nucleic acid testing sites to facilitate rapid testing and curb outbreaks. However, these sites often experience congestion, increasing transmission risks and reducing testing efficiency. This study focuses on the spatial–temporal analysis of testing site distribution and associated infection risks in Shenzhen, China. Data from all Shenzhen testing sites were analyzed for the week of October 24–30, 2022, noting the percentage of busy hours per site and incorporating a population size factor by district to assess regional infection risks. Findings indicate three daily peak testing times—primarily in the evening—with the highest risk of transmission in Longgang District, followed by Yantian and Luohu, and the lowest in Futian. The risk coefficient varied from 0.040 to 0.349, with most areas showing stable risk levels between 0.06 and 0.20. This research underlines the necessity for policymakers to alleviate congestion at testing sites and suggests increasing site availability in Longgang District to mitigate COVID-19 spread, offering methodological guidance for managing infection risks in other major Chinese cities.

## 1 Introduction

Since the December 2019 outbreak of Severe Acute Respiratory Syndrome Coronavirus 2 (SARS-CoV-2) widely spread to the world, caused Corona Virus Disease 2019 (COVID-19) (1). The spread of the virus has been accelerated by continued rapid genetic mutations, which have placed a serious burden on the public health care sector in various countries. The effectiveness of the traditional method of administering COVID-19 vaccine to citizens to slow the spread of the virus and clinical symptoms has been widely demonstrated (2, 3). However, we still need to look for other environmental factors for virus transmission. Several studies have shown that the urban built environment factors of public transportation, points of interest and recreational spaces, areas where people congregate, are thought to be positively associated with COIVD-19 transmission (4–6). Crowd aggregation is also a concern, and the extremely high population density of mega-cities in various countries can increase the spread of COVID-19 between populations (7, 8). A negative relationship between the number of COVID-19 infection cases and urban population mobility has been demonstrated in several studies (8). Therefore, in order to combat the spread of COVID-19 in cities and reduce the number of illnesses and deaths, many countries are actively taking various non-pharmaceutical measures in addition to vaccinating their citizens to create basic immunity. Examples include urban lockdowns, home isolation, close access control, and high frequency nucleic acid testing (9). As in the early stages of the outbreak, the influx of patients caused a run on medical resources. Therefore, there is a need for nucleic acid testing measures at the residential community level to identify infected individuals as early as possible (10). Transmission risk factors are influenced by multiple elements, including a high proportion of older adult population, economic difficulties, high population density, low socioeconomic status, and unique geographical locations. The combined effect of these risk factors impacts the transmission and mortality rates of COVID-19, significantly affecting the spatial–temporal dynamics of the pandemic (11).

**Figure 1:**
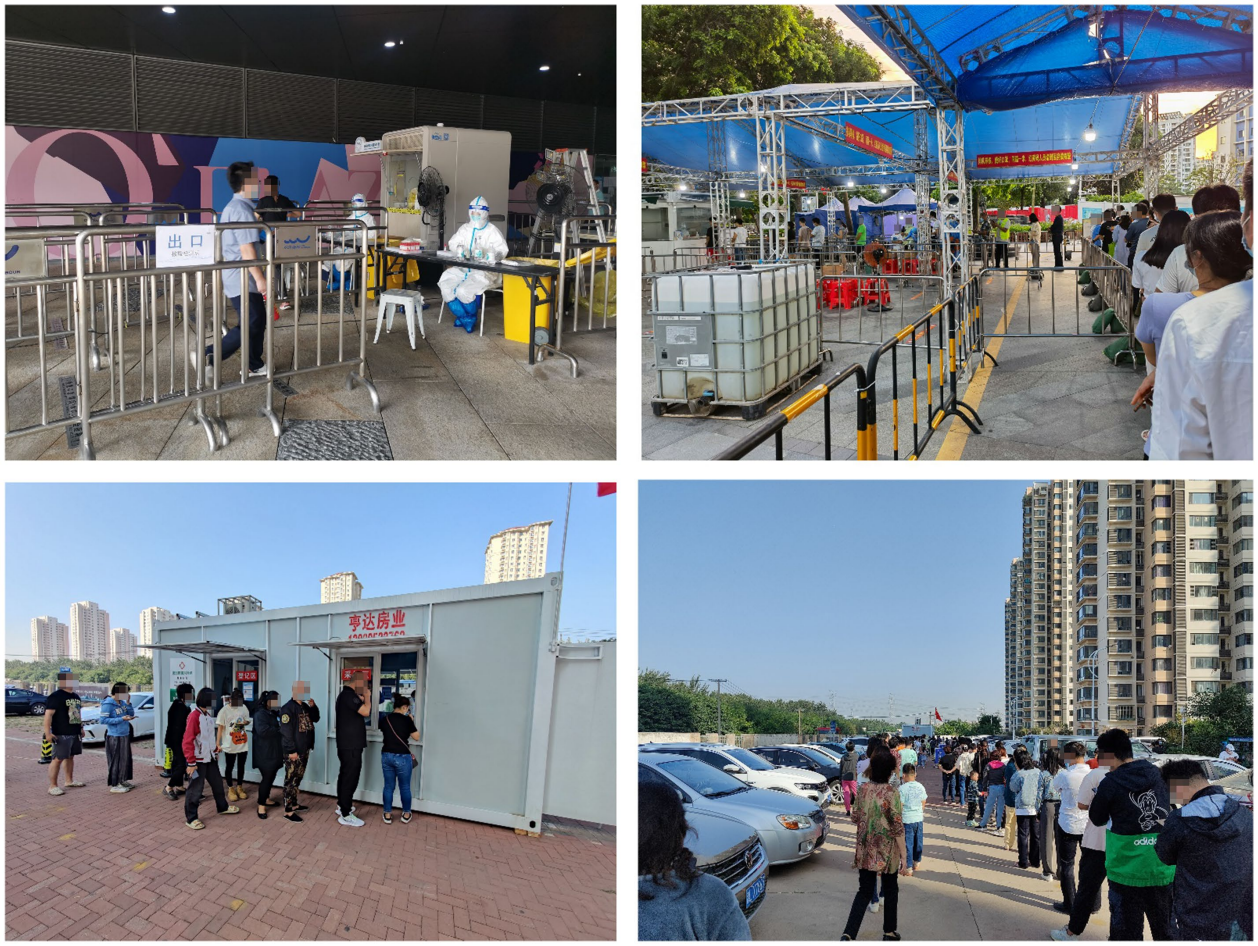
China COVID-19 pneumonia nucleic acid test site queue.

The concept of a 15 min living circle was introduced to facilitate the quick and easy completion of nucleic acid testing for residents and is strongly supported by the Chinese government (12). This means that every resident in the city can have a COVID-19 nucleic acid test within 15 min, which is not an easy task for a country with a large population like China. Such a high frequency of testing of the population will increase the efficiency of early detection of cases. This requires the installation of a nucleic acid testing site within a residential area, which is a temporary medical infrastructure (Figure 1) with several features. (1) It provides a safe working place for the Nucleic Acid Testing staff, separated from the population. (2) The mobile nature of the nucleic acid testing site is also a key feature, which makes it easier to move to more crowded locations. (3) The necessary disinfection measures, such as UV disinfection or air purification systems, are in place. According to research data, few studies have been conducted to identify the risk of virus transmission by exploring the busyness of nucleic acid testing sites. There is a strong correlation between waiting times at nucleic acid testing sites and the spread of the virus, and a direct relationship with the well-being of the population. In this regard, Shenzhen, Guangdong Province, a typically densely populated Asian city, is an ideal site. Identifying the busyness and spatial distribution of nucleic acid testing sites can help urban decision makers to plan the placement of nucleic acid testing sites in a rational way, thus providing data to effectively reduce queuing times and transmission risks.

## 2 Materials and methods

### 2.1 Target area

The study area is selected from one of the most economically developed cities in China, Shenzhen, Guangdong Province. According to the latest Chinese census data Shenzhen has a total population of approximately 17 million and a high population density. Therefore, a study on the busyness of the nucleic acid testing sites in this city can reduce the queuing time to reduce the risk of COVID-19 transmission. The climate has an average year-round temperature of 22.4°C and a southern subtropical monsoon climate. The mild and comfortable climate provides a basis for excluding weather factors from influencing the distribution of nucleic acid testing sites. The commercial center of the city is concentrated in the southern plain area. The rest of the residential areas and service settings are evenly distributed in the surrounding areas. Shenzhen has 13 subway lines passing through the city, which also greatly enhances the accessibility of the region, making it easier for people to travel long distances and reach the nearest nucleic acid testing sites in a short time, which also allows the study to be explored from multiple perspectives.

### 2.2 COVID-19 nucleic acid detection sites

Since the establishment of China’s regular epidemic control measures, many cities have built a large number of COVID-19 nucleic acid testing sites to facilitate rapid nucleic acid testing for residents. In Shenzhen, real-time information on all testing sites has been aggregated in the form of a smart city and made available on a unified website, so that all residents can check the status of the sites at any time.

Our research obtained real-time information on nucleic acid testing sites through this project website.^1^ We collected nucleic acid test site status information 24 h a day for 7 days from 24 October 2022 to 30 October 2022, with no Chinese holidays, to avoid the impact of holidays on the busyness of the tests. Each test site has a very large number of real-time statuses (Table 1), and we filtered out a few pieces of information needed for the study objectives to be retained.

**Table 1:** Sampling data sheet for nucleic acid testing sites.

Data name	Example data	Data interpretation
Address	Yunfeng Technology Building (Songping Street East), Nanshan District, Shenzhen, Guangdong Province	Information on the location of nucleic acid sampling sites
Area name	Nanshan District	Nucleic acid sampling sites in the administrative districts of Shenzhen
Code	B04103712	Unique identification number according to administrative division
Id	108551	Unique identification number
Latitude	22.555162	Latitude of nucleic acid sampling sites
Longitude	113.956486	Longitude of nucleic acid sampling sites
Status	3	Nucleic acid sampling site busy status

This research has designed a specific process to deal with complex data structures, including the following steps:

1. Data collection. Data is logged hourly using the Python program’s request library for nucleic acid test site data;
2. Logging the data. After obtaining the accessed data, the data is presented in json format and we save the required information in csv format;
3. Cleaning the data. We use the pandas library to filter the data and keep the ones that have data distribution within 24 h;
4. Processing the data. The definitions of these four states are provided by the website, and we use 0, 1, 2, and 3 to represent these four states. The status of the data represents the busy status of the nucleic acid testing sites, where 1 represents rest (closed), 2 represents open (queue less than 15 min), 3 represents busy (queue 15–30 min) and 4 represents congested (queue greater than 30 min). The pandas library was used to filter the percentage of statuses 3 and 4 in the 24 h period to represent the percentage of busy nucleic acid detection sites.

### 2.3 Nucleic acid testing site busyness and calculation of infection risk factors

First, for a single nucleic acid detection site, 24 observations will be recorded each day, and each observation will have four states possible, and we define the number of times these four states occur in a day as *s*1, *s*2, *s*3, and *s*4, so for a 24 h period on date *i*, we can calculate the percentage of hours with queuing times greater than 15 min at the nucleic acid detection site in a single day (*B*), calculated (1) as:


(1) $$B_{i}=\frac{s3_{i}+s4_{i}}{s1_{i}+s2_{i}+s3_{i}+s4_{i}}$$

The average percentage of nucleic acid testing sites that are busy on a single day in a borough (*D*), which accurately reflects the busyness of testing sites over the course of a day, where n is the number of nucleic acid sites in the borough, is calculated (2) as:


(2) $$D_{i}=\frac{1}{n}\overset{n}{\underset{i=1}{\sum }}B_{i}$$

The average percentage of busy nucleic acid testing sites (*W*) for a week in the administrative area was counted to accurately reflect how busy the sites were over the course of the week, where d is the number of days observed in this study and is calculated (3) as:


(3) $$W_{i}=\frac{1}{d}\overset{d=7}{\underset{i=1}{\sum }}D_{i}$$

To measure the risk factor (*F*) of infection during the COVID-19 test queue, we introduced the population size *P* (in 10,000 people) per administrative district to correct for this factor. In which *W* represents the average busyness ratio of the nucleic acid testing points over a week, as described in Equation (3), *n* is the number of nucleic acid testing sites in the administrative district, and *i* denotes the different administrative districts of Shenzhen, the formula was calculated (4) as:


(4) $$F_{i}=\frac{P_{i}}{n}\times W_{i}$$

## 3 Results

### 3.1 Busy status of COVID-19 nucleic acid detection sites in Shenzhen

**Figure 2:**
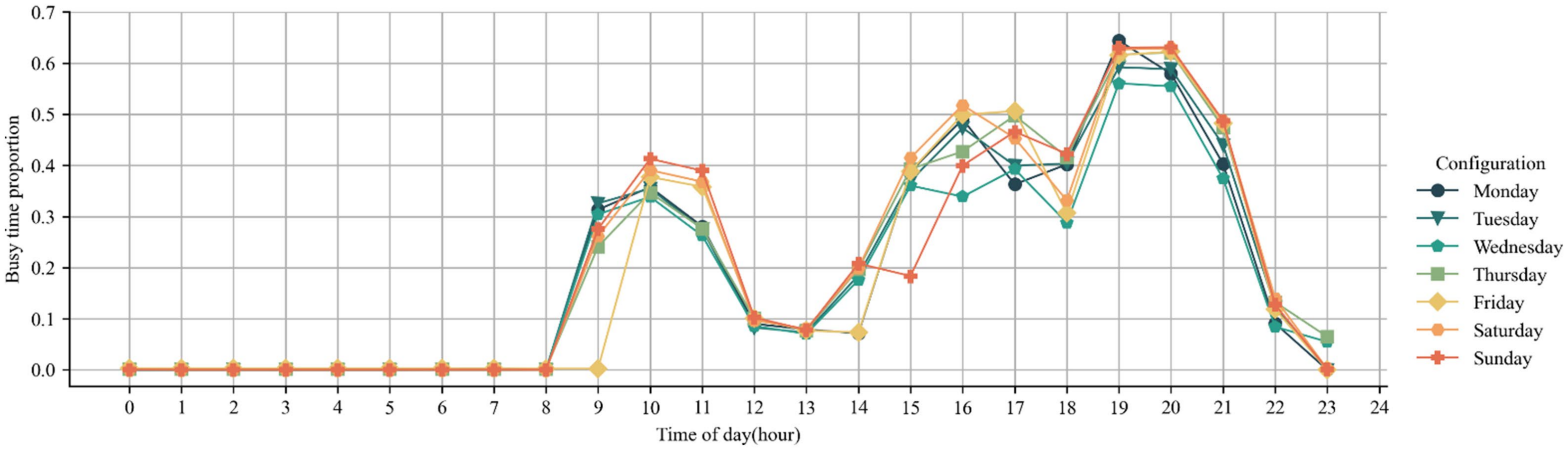
Folding line graph of the average 24 h busy distribution at the test site.

We recorded the busy status of all nucleic acid testing sites for 24 h per day over a one-week time frame. We counted the number of nucleic acid testing sites in Shenzhen with queue times greater than 15 min per hour and calculated their share of all nucleic acid testing sites. In Figure 2, we plot a line graph of the percentage of busy nucleic acid sites for a week. The results of this graph show that there are three peak hours in the city for residents to have nucleic acid tests: 9–11 am, 13–17 pm and 19–21 pm. The number of busy nucleic acid testing sites during these three periods is approximately 40, 50 and 60% respectively, showing an upward trend, which means that people in the city are more likely to have their nucleic acid tests done in the evening, followed by midday and morning. It is also interesting to note that during the morning rush hours on Thursday, Friday and Saturday, the highest percentage of busy nucleic acid users arrived 1 h later than at other times, and the percentage of busy users was also higher. This suggests that on weekends, residents generally delay their nucleic acid tests by 1 h compared to weekdays, and that the busy rate falls to 10% at 12:00 pm on weekends as well as on weekdays, so that crowds are more concentrated on weekends.

**Figure 3:**
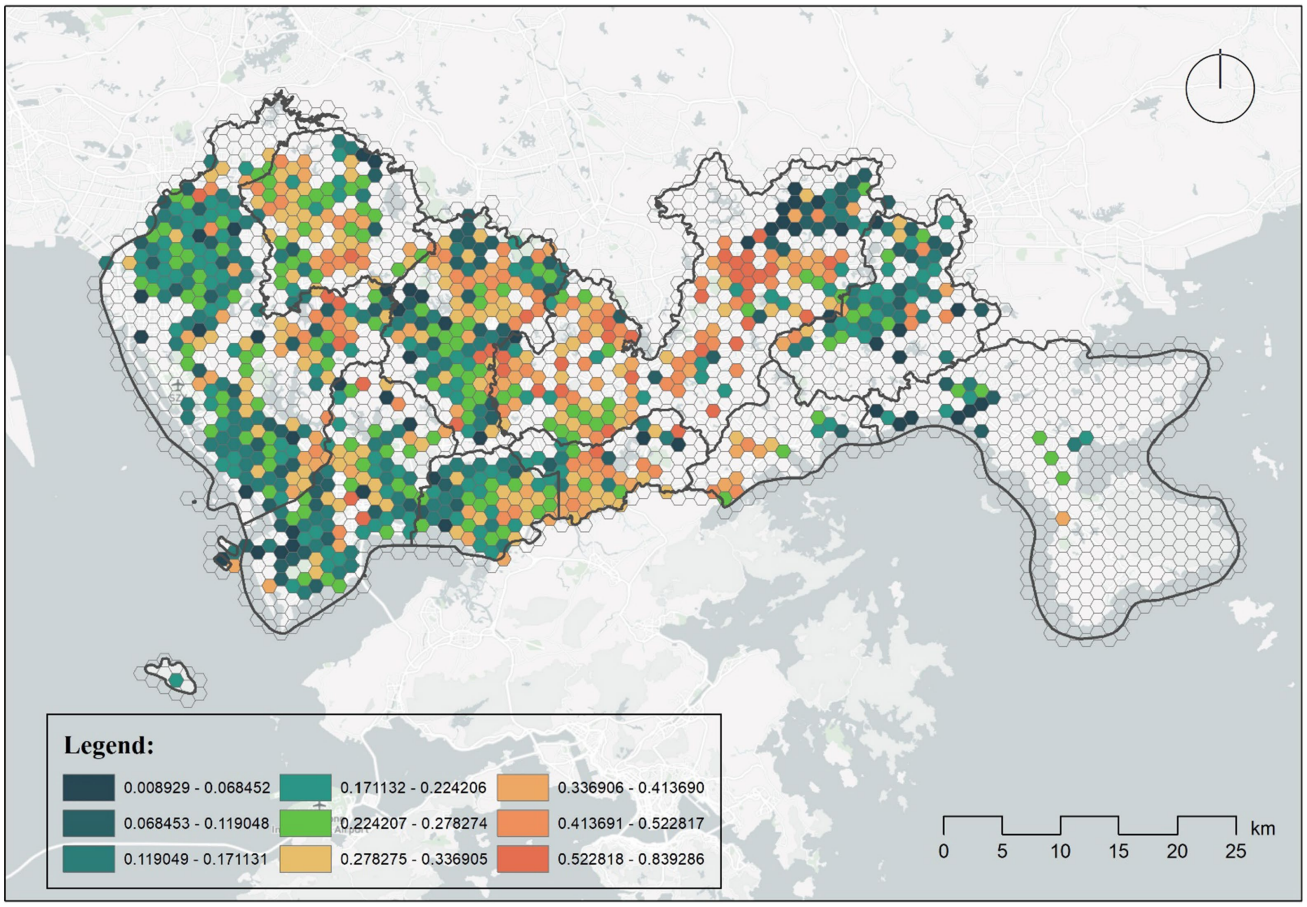
Average busy distribution of testing sites over a week.

We calculated the percentage of busy nucleic acid testing sites for a week and based on this plotted Figure 3 depicts the geographic distribution of sites that are more likely to be in queues. We defined busy nucleic acid testing sites as those with queues of 15 min or more for more than 40% of the day, and such sites were mainly located in Longgang District, with some long queues in Luohu District, Bao’an District and Nanshan District. Other areas such as Nanshan District, Pingshan District and Bao’an District have a lower percentage of busy sites.

### 3.2 Risk factors for COVID-19 infection by administrative districts in Shenzhen

**Figure 4:**
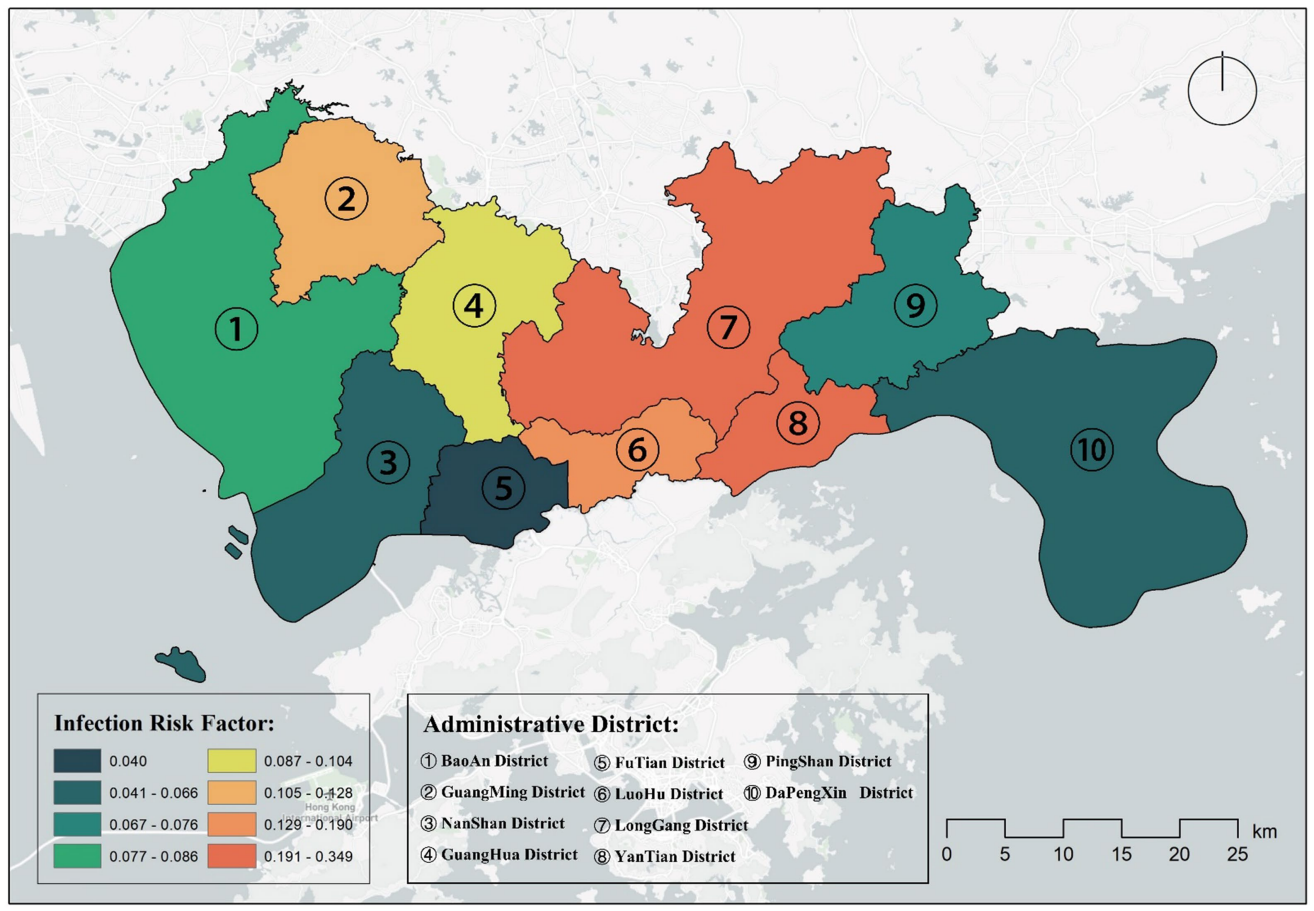
Distribution of administrative districts at risk of COVID-19 infection in Shenzhen.

Data on the number of people in the administrative area were obtained from the seventh census data,^2^ and we calculated and presented in Table 2 by considering several factors such as population factors, the number of nucleic acid testing sites and the proportion of busy people. The risk factors for COVID-19 infection are shown in Table 2. We plotted the distribution of COVID-19 risk factors in geographical space in Figure 4, showing that the highest risk of infection was found in Longgang and Yantian districts, followed by Guangming and Luohu districts, which were also at high risk of COVID-19 infection. On the other hand, the areas at lower risk of COVID-19 infection were Nanshan District, Futian District, Pingshan District and Dapeng New District. The results showed that the risk factor for COVID-19 infection was highly correlated with the proportion of busy nucleic acid testing sites, with the busier nucleic acid testing sites being more likely to generate population clusters and cause the spread of COVID-19 virus, again in line with our expected hypothesis.

**Table 2:** Risk factors for COVID-19 infection in Shenzhen.

Administrative District	Number of nucleic acid testing sites	Number of residents served per nucleic acid sites (10,000)	Average percentage of busy week (%)	COVID-19 risk factor for infection
Futian District	818	0.190	0.208	0.040
Luohu District	178	0.643	0.296	0.190
Yantian District	22	0.974	0.318	0.310
Nanshan District	572	0.314	0.195	0.061
Bao’an District	1,091	0.410	0.209	0.086
Longgang District	355	1.121	0.311	0.349
Longhua District	560	0.452	0.231	0.104
PingShan District	142	0.389	0.196	0.076
Guangming District	247	0.443	0.289	0.128
Dapeng New District	34	0.460	0.144	0.066

In Table 3, we provide a statistical description of population characteristics such as population size, population density, and number of communities for each administrative district in Shenzhen. We observe that although Futian District has the highest population density, its infection risk coefficient is relatively low (0.040). In contrast, Longgang District, despite not having the highest population density, has the highest infection risk coefficient (0.349). The number of communities can be seen as an indicator of the distribution of residents and the development of infrastructure in an area. Generally, a higher number of communities implies more residential areas and more intensive population activities. For example, Bao’an District has the most communities (124) and the largest population, with an infection risk coefficient at a medium level (0.086). This reflects that even though there are many communities, adequate testing sites can effectively control the infection risk. The area of the administrative district is also an important factor. Dapeng New District has the largest area but an extremely low population density, and its infection risk coefficient is also relatively low (0.066).

**Table 3:** Population characteristics by administrative region in Shenzhen.

Administrative District	Population of administrative district (10,000)	Administrative area (km^2^)	Density of population (10,000/km^2^)	Number of communities
Futian District	155.3225	78.66	1.9746	92
Luohu District	114.3801	78.79	1.4517	81
Yantian District	21.4225	74.99	0.2857	19
Nanshan District	179.5826	185.22	0.9696	101
Bao’an District	447.6554	397	1.1276	124
Longgang District	397.9037	388.21	1.0250	111
Longhua District	252.8872	175.6	1.4401	69
PingShan District	55.1333	166	0.3321	23
Guangming District	109.5289	156.1	0.7017	31
Dapeng New District	15.6236	600	0.0260	25

### 3.3 Increasing nucleic acid testing sites to reduce transmission risk

By utilizing data on the number of nucleic acid testing sites and the populations they serve, we have calculated the infection risk coefficient. The mean infection risk coefficient was determined to be 0.141. Our aim is to lower the infection risk coefficient in the three administrative districts with the highest coefficients to below this mean by increasing the number of nucleic acid testing sites. Consequently, we have derived the necessary increase in testing sites required to meet this goal.

As shown in Table 4, Luohu District, Yantian District, and Longgang District have infection risk coefficients above the mean. Thus, these districts should be prioritized for the addition of testing sites. Our calculations indicate that Luohu District, Yantian District, and Longgang District need to increase their nucleic acid testing sites by 240, 48, and 877 respectively, to bring their infection risk coefficients down to the average. This represents increases of 34.90, 119.61, and 147.22% relative to their current number of testing sites. Since these figures are derived from simulations, the actual numbers must be adjusted based on regional circumstances, population distribution, and the actual levels of infection.

**Table 4:** The risk of infection is reduced to mean (0.141) costing.

Administrative District	COVID-19 risk factor for infection	Mean difference	Increased quantity of number of residents served per nucleic acid sites (10,000)	Increased of number of nucleic acid testing sites	Percentage increase of nucleic acid test sites
Futian District	0.040	−0.101			
Luohu District	0.190	0.049	0.476	240.117	+34.90%
Yantian District	0.310	0.169	0.443	48.315	+119.61%
Nanshan District	0.061	−0.08			
Bao’an District	0.086	−0.055			
Longgang District	0.349	0.208	0.453	877.646	+147.22%
Longhua District	0.104	−0.037			
PingShan District	0.076	−0.065			
Guangming District	0.128	−0.013			
Dapeng New District	0.066	−0.075			

## 4 Discussion

### 4.1 The research findings

The current viral pandemic poses new challenges for national and regional urban planning. How to make our countries, regions and cities resistant to viruses has been a hot topic of discussion in various disciplines. This study provides a method to determine the risk of COVID-19 transmission due to crowded queues, using data from an unnoticed COVID-19 nucleic acid testing site that emerged during an epidemic, in terms of geospatial location of the nucleic acid testing site, queueing time status, and continuous long-term observed status changes. This study has important practical implications for the study of the relationship between the density of nucleic acid testing sites and the risk of COVID-19 infection, and represents a new starting perspective. High frequency of COVID-19 nucleic acid screening is an effective response to epidemics, but the efficient allocation of public resources is an important issue in a context of resource constraints in public health systems in different regions.

### 4.2 Epidemic prevention and control policy

Based on the research findings, we can observe that the spatial and temporal distribution of nucleic acid testing sites in Shenzhen has revealed significant risk factors for COVID-19 transmission. The study found that during specific peak periods, such as evenings and weekends, the risk of transmission increased due to crowd gatherings and prolonged waiting times for testing. These high-risk periods were mainly concentrated in Longgang and Yantian districts, while the risk in Futian district was relatively lower. This situation indicates that the government faces significant challenges in managing the pandemic and controlling its spread in certain areas. To address these challenges, the government can implement the following strategies:

1. Increase the number of nucleic acid testing sites: Especially in densely populated areas and during high-risk periods, adding more testing sites can reduce waiting times and thereby decrease the chances of crowd gatherings.
2. Optimize the time management of testing sites: Adjust the operating hours of certain sites based on data analysis, particularly during evenings and weekends, to disperse peak traffic.
3. Improve testing efficiency: Enhance the operational efficiency of testing sites by introducing faster testing technologies and more efficient data processing systems. Alternatively, increase the staffing levels at individual testing points to boost testing capacity.
4. Public education and communication: The behavior and response of individuals and communities are crucial in managing infection risks. Strengthening public education on pandemic prevention, clearly communicating the optimal testing times and site choices, avoiding testing during peak periods, actively responding to government health guidance, and adopting personal protective measures are all key factors in reducing infection risk. Therefore, raising public awareness and understanding of these risk factors, and how individual actions can mitigate these risks, is an essential component of improving public health response capabilities.
5. Use of Geographic Information Systems (GIS): Utilize GIS technology to monitor the flow of people and the spatial distribution of testing sites in real time, allowing for rapid adjustment of resource allocation and response strategies.

By implementing these strategies, the government can manage public health resources more effectively, reduce the risk of COVID-19 transmission, and ensure the normal conduct of socio-economic activities. These strategies not only apply to current pandemic management but also provide policy and operational references for potential future public health events.

### 4.3 Limitation and future works

This study has certain limitations. While it examines the spatial and temporal distribution characteristics of the busyness of nucleic acid testing sites, it does not explore the influencing factors behind this busyness. The busyness of COVID-19 testing sites may be influenced by a range of external factors, including demographic characteristics (such as age structure, health status, and vaccine coverage), preventive measures (such as wearing masks, maintaining social distancing, and disinfection practices), geographical environmental factors (such as urban road accessibility, land use attributes, and current regional economic development), and the availability of community resources to mitigate risks. Additionally, the busyness of COVID-19 testing sites constitutes geographical data, which may exhibit spatial correlation, meaning that testing sites at different distances could influence each other. There might also be some heterogeneity in busyness levels, indicating that the influencing factors of the busyness of nucleic acid testing sites may have different effects in different regions.

Future research should not only investigate the factors influencing the busyness of nucleic acid testing sites but also incorporate the spatial heterogeneity effects of these influencing factors. Furthermore, actively collaborating with the government to obtain data samples of nucleic acid testing sites over a broader timeframe would significantly enhance the accuracy of the research. Overall, this study pioneeringly proposes a new method to measure COVID-19 transmission risk based on the busyness and number of nucleic acid testing sites. We believe that the method proposed in this study will have a long-term impact on urban planning and policy-making.

## Data availability statement

The original contributions presented in the study are included in the article/supplementary material, further inquiries can be directed to the corresponding authors. The collected source data, the analysis result data, and all processing source code are available at: https://doi.org/10.6084/m9.figshare.21499236.v1.

## Author contributions

LW: Data curation, Formal analysis, Methodology, Project administration, Writing – original draft, Writing – review & editing. LZ: Formal analysis, Validation, Visualization, Writing – original draft, Writing – review & editing. TZ: Funding acquisition, Methodology, Project administration, Supervision, Writing – original draft, Writing – review & editing. XH: Writing – original draft.

## Footnotes

^1^https://hsdyjc.newhealth.com.cn/wh5/#/

^2^http://tjj.sz.gov.cn/ztzl/zt/szsdqcqgrkpc/szrp/content/post_8772114.html

## References

[1] Guan W Ni Z Hu Y Liang W Ou C He J. Clinical characteristics of coronavirus disease 2019 in China. N Engl J Med. (2020) 382:1708–20. doi: 10.1056/NEJMoa2002032, PMID: 32109013 PMC7092819

[2] Polack FP Thomas SJ Kitchin N Absalon J Gurtman A Lockhart S. Safety and efficacy of the BNT162b2 mRNA COVID-19 vaccine. N Engl J Med. (2020) 383:2603–15. doi: 10.1056/NEJMoa2034577, PMID: 33301246 PMC7745181

[3] Wood H. Safety and efficacy of COVID-19 vaccines in people with neurological disorders. Nat Rev Neurol. (2022) 18:66. doi: 10.1038/s41582-021-00603-8, PMID: 34887547 PMC8655711

[4] Figueroa JF Wadhera RK Mehtsun WT Riley K Phelan J Jha AK. Association of race, ethnicity, and community-level factors with COVID-19 cases and deaths across U.S. counties. Healthcare. (2021) 9:100495. doi: 10.1016/j.hjdsi.2020.100495, PMID: 33285500 PMC7680060

[5] Liu C Liu Z Guan C. The impacts of the built environment on the incidence rate of COVID-19: a case study of King County, Washington. Sustain Cities Soc. (2021) 74:103144. doi: 10.1016/j.scs.2021.103144, PMID: 34306992 PMC8271037

[6] Wang L Zhang S Yang Z Zhao Z Moudon AV Feng H. What county-level factors influence COVID-19 incidence in the United States? Findings from the first wave of the pandemic. Cities. (2021) 118:103396. doi: 10.1016/j.cities.2021.103396, PMID: 34334868 PMC8316070

[7] Balbontin C Hensher DA Beck MJ Giesen R Basnak P Vallejo-Borda JA. Impact of COVID-19 on the number of days working from home and commuting travel: a cross-cultural comparison between Australia, South America and South Africa. J Transp Geogr. (2021) 96:103188. doi: 10.1016/j.jtrangeo.2021.103188, PMID: 34493910 PMC8413364

[8] Chan HF Brumpton M Macintyre A Arapoc J Savage DA Skali A. How confidence in health care systems affects mobility and compliance during the COVID-19 pandemic. PLoS One. (2020) 15:e0240644. doi: 10.1371/journal.pone.0240644, PMID: 33057450 PMC7561184

[9] Sharifi A Khavarian-Garmsir AR. The COVID-19 pandemic: impacts on cities and major lessons for urban planning, design, and management. Sci Total Environ. (2020) 749:142391. doi: 10.1016/j.scitotenv.2020.142391, PMID: 33370924 PMC7499053

[10] Wang J. Vision of China’s future urban construction reform: in the perspective of comprehensive prevention and control for multi disasters. Sustain Cities Soc. (2021) 64:102511. doi: 10.1016/j.scs.2020.102511, PMID: 33014695 PMC7518975

[11] Sartorius B Lawson AB Pullan RL. Modelling and predicting the spatio-temporal spread of COVID-19, associated deaths and impact of key risk factors in England. Sci Rep. (2021) 11:5378. doi: 10.1038/s41598-021-83780-2, PMID: 33686125 PMC7940626

[12] Weng M Ding N Li J Jin X Xiao H He Z. The 15-minute walkable neighborhoods: measurement, social inequalities and implications for building healthy communities in urban China. J Transp Health. (2019) 13:259–73. doi: 10.1016/j.jth.2019.05.005

